# *Neisseria gonorrhoeae* Multivalent Maxibody with a Broad Spectrum of Strain Specificity and Sensitivity for Gonorrhea Diagnosis

**DOI:** 10.3390/biom11030484

**Published:** 2021-03-23

**Authors:** Jieun Jeong, Jae-Seok Kim, Junghyeon Lee, Yu Ri Seo, Eugene C. Yi, Kristine M. Kim

**Affiliations:** 1 Division of Biomedical Convergence, College of Biomedical Science, Kangwon National University, Chuncheon, Gangwon 24341, Korea; jje7442@kangwon.ac.kr; 2 Department of Laboratory Medicine, Kangdong Sacred Heart Hospital, Gangdong-gu, Seoul 05355, Korea; jaeseok@hallym.ac.kr; 3 Department of Laboratory Medicine, College of Medicine, Hallym University, Chuncheon 24341, Korea; 4 Department of Bio-Health Convergence, Kangwon National University, Chuncheon 24341, Korea; sunshine2960@kangwon.ac.kr; 5 Department of Molecular Medicine and Biopharmaceutical Sciences, Graduate School of Convergence Science and Technology, College of Medicine or College of Pharmacy, Seoul National University, Seoul 03080, Korea; yuri.seo@snu.ac.kr

**Keywords:** *Neisseria gonorrhoeae*, multivalent maxibody, diagnosis, immunodiagnostics, bio-panning, sexually transmitted disease (STD), infectious disease, antibody

## Abstract

Gonorrhea is one of the most common, but still hidden and insidious, sexually transmitted diseases caused by *Neisseria gonorrhoeae* (*gonococci*). However, the diagnosis and treatment of gonorrhea are hampered by antigenic variability among *gonococci*, the lack of acquired immunity, and antimicrobial resistance. Further, strains resistant to cephalosporins, including ceftriaxone, the last line of defense, represent a growing threat, which prompted us to develop *gonococci*-specific diagnostic antibodies with broad-spectrum binding to *gonococci* strains to generate gonorrhea-detecting reagents. This study reports the identification of *gonococci* antibodies via bio-panning on *gonococci* cells using scFv-phage libraries. Reformatting the lead scFv-phage Clones 1 and 4 to a multivalent scFv1-Fc-scFv4 maxibody increased the sensitivity by up to 20-fold compared to the single scFv-Fc (maxibody) alone. Moreover, the multivalent maxibody showed broader cross-reactivity with clinical isolates and the ceftriaxone antibiotic-resistant World Health Organization (WHO) reference strain L. In contrast, the selected antibodies in the scFv-phage, maxibody, and multivalent maxibody did not bind to *N. sicca*, *N. meningitides*, and *N. lactamica*, suggesting the clinical and pharmaceutical diagnostic value of these selected antibodies for gonorrheal infections. The present study illustrates the advantages and potential application of multivalent maxibodies to develop rapid and sensitive diagnostic reagents for infectious diseases and cancer.

## 1. Introduction

Gonorrhea is a sexually transmitted infectious disease (STI/STD) caused the by gram-negative pathogen *Neisseria gonorrhoeae*, which usually occurs as diplococci [1]. *Neisseria* species belong to a large genus of bacteria that colonizes the mucosal surfaces of many animals. Of the 13 *Neisseria* species that colonize humans, only *N. meningitidis* and *N. gonorrhoeae* are pathogenic [2]. Gonorrhea is one of the major public health priorities globally due to the antimicrobial resistance and hypervariability of the *N. gonorrhoeae* strain. In 2016, the World Health Organization (WHO) estimated nearly 87 million gonorrheal infection cases among adults worldwide [3]. A 67% increase in gonorrhea rates since 2013 in the United States and a global increase in infections among homosexual and bisexual men remains a global public health concern [4,5,6]. While uncomplicated gonococcal infections commonly manifest as urethritis in men with urethral discharge and dysuria, severe reproductive complications including urethral and genital discharge, infertility, pelvic inflammatory disease, and ectopic pregnancy in women can result from the absence of prompt diagnosis and treatment [7,8,9].

Moreover, the widespread multi-drug resistant (MDR) variants of *N. gonorrhoeae* strains have escalated globally and thus compromised the management and control of gonorrhea in recent years [10,11]. Resistance to ceftriaxone, which is a third-generation antibiotic and the last gonorrheal treatment option available as the first-line monotherapy, has been reported worldwide [6,12,13]. Curative therapy is currently achievable with a dual-therapy regimen of ceftriaxone and azithromycin antibiotics. Recently, ertapenem has rapidly emerged as a gonorrhea treatment due to multidrug-resistant gonorrhea [14,15]. However, gonorrheal treatment failure with dual antimicrobial therapy (ceftriaxone combined with azithromycin or doxycycline) has been confirmed worldwide, as well [16,17,18,19,20]. Thus, the limited treatment options and the spread of hyper-variant ceftriaxone and azithromycin-resistant *N. gonorrhoeae* strains are significant public health concerns.

Therefore, clinical testing and treatment options matching the evolving pace of *N. gonorrhoeae*-resistant strains are needed. Consequently, the development of new drugs, alternative therapies or vaccines, and rapid and accurate diagnosis of infections is essential for the adequate control of *N. gonorrhoeae* [3,14].

Accurate identification of populations of individuals with the disease is essential in diagnostics. Poor sensitivity for *N. gonorrhoeae* and cross-reactivity with other *Neisseria* species are limitations of the traditional diagnostic methods for gonorrhea via Gram staining, bacterial culture, and immunochemistry [21,22]. *Polymerase chain reaction* (PCR)-based nucleic acid amplification tests (NAATs) for *N. gonorrhoeae* significantly improved both the sensitivity and specificity of gonorrhea diagnosis [22]. Currently, Io CT/NG^®^ by Binx Health and GenXpert CT/NG^®^ by Cepheid are the only FDA-approved NAATs for gonorrhea [23,24]. However, these tests require controlled and restricted laboratory environments for diagnostic evaluation, with results generally available in a few days and are expensive to perform in developing countries. In contrast, ASSURED criteria established by the WHO for developing new STD diagnostics require that the point-of-care tests (POCTs) be affordable, sensitive, specific, user-friendly, rapid and robust, equipment-free, and deliverable to end-users. Consequently, the FDA-approved NAATs for gonorrhea are not utilized widely as POCTs due to the failure to meet the criteria for STD diagnostics.

In contrast, POCTs based on immunological methods (i.e., immunodiagnostics) still need to overcome issues associated with poor sensitivity and specificity due to the antigenic variability of *N. gonorrhoeae* strains for the detection of gonorrhea infections while meeting the established criteria [23,24,25,26,27,28,29]. These issues are also reflected, in part, in commercial *N. gonorrhoeae* antibodies, which show poor specificity or the failure to recognize their targets. For example, the sensitivity for detecting *N. gonorrhoeae*-positive female specimens was 70%, 12.5%, and 60% using GC Check (PATH), ACON NG Duo tests (ACON Laboratories, San Diego, USA), and Biostar OIA GC (ThermoFisher Scientific, Waltham, USA) test kits in development, respectively, compared with the standard reference tests confirmed by NAAT or culture tests [28,30,31]. Furthermore, the confidence interval (CI) for identifying *N. gonorrhoeae* in clinical specimens ranged between 55% and 82%, 0 and 41.7%, and 46 and 74% for GC Check, ACON NG Duo tests, and Biostar OIA GC, respectively [24]. Consequently, FDA-approved immunodiagnostic tests such as POCTs and enzyme-linked immunosorbent assays (ELISAs) for gonorrhea are unavailable. Therefore, the availability of improved *N. gonorrhoeae*-specific antibodies with high sensitivity can overcome the challenges associated with developing POCTs and controlling gonorrheal infections via early detection, including STD-related stigma, which prevents individuals from seeking treatment and screening for infection.

Thus, we were inspired to develop *N. gonorrhoeae*-specific broad-spectrum antibodies against the diverse strains of *N. gonorrhoeae* to generate improved sensitivity and specificity compared to the currently available antibody reagents for POCTs. Since traditional immunological methods using monoclonal antibodies (mAbs) for *N. gonorrhoeae* have failed to provide satisfactory diagnostic results, we considered a bispecific-like antibody as an alternative approach to improve the sensitivity and specificity of *N. gonorrhoeae* detection. Bispecific antibodies (bsAbs) combine the specificity of two antibodies and thus, simultaneously bind two different antigens or epitopes. The stability and functionality of various bsAb platforms have led to clinical trials and approval for therapy [32,33,34,35]. For example, MM141 (anti-HER3 and anti-IGF1R) is a bsAb with scFvs genetically fused at the C-terminus of an IgG [36]. This bsAb is a tetravalent mAb, which contains a total of four target binding sites, two for each specific target, and is currently undergoing a phase 2 trial in patients with metastatic pancreatic cancer [36,37].

Therefore, we wanted to generate antibodies with multiple binding sites specific for *N. gonorrhoeae* (i.e., a multivalent antibody) for application in immunodiagnostics. The conversion of mAbs into the multivalent maxibody form, scFv-Fc-scFv, was expected to enhance the binding to patient-derived *N. gonorrhoeae* and reduce cross-reactivity to non-gonorrhea-related *Neisseria* species. In this study, we present the generation of *N. gonorrhoeae*-specific multivalent maxibodies to investigate the specificity and sensitivity of the antibodies selected by cell-based bio-panning. Our results suggest that the newly developed *N. gonorrhoeae* multivalent maxibody may serve as a starting material to facilitate diagnosis with enhanced sensitivity and specificity for the detection of *N. gonorrhoeae*.

## 2. Materials and Methods

### 2.1. Neisseria gonorrhoeae Cell Culture

*N. gonorrhoeae* clinical isolates were obtained from Kangdong Sacred Heart Hospital and cultured on chocolate agar plates (Shin Yang Chemical, Busan, Korea) at 37 °C under 5% CO_2_. In-vitro cell-based bio-panning was performed by scraping and suspending the colonies on the plates in phosphate-buffered saline (PBS). The bacterial suspension was heat-inactivated for 30 min at 56 °C. The strains of *N. meningitidis*, *N. sicca*, and *N. lactamica* were used as negative controls. *N. meningitides* was obtained from the Korea Centers for Disease Control and Prevention, and *N. sicca* (ATCC9913) and *N. lactamica* (ATCC23970) were purchased from the American Type Culture Collection.

### 2.2. Characterization of N. gonorrhoeae Clinical Isolates

The molecular clonality of 11 isolates was analyzed for NG-MAST typing and PubMLST typing according to the method we previously reported [38]. Antimicrobial susceptibility testing was performed and interpreted according to the Clinical and Laboratory Standards Institute (CLSI) M100-S25 document using standard disc diffusion methods [38,39]. Eight isolates were tested for β-lactamase production using nitrocefin solution (Oxoid™, Waltham, MA, USA) as described by the WHO [40].

### 2.3. In-Vitro Cell-Based Bio-Panning for N. gonorrhoeae

**Figure 1:**
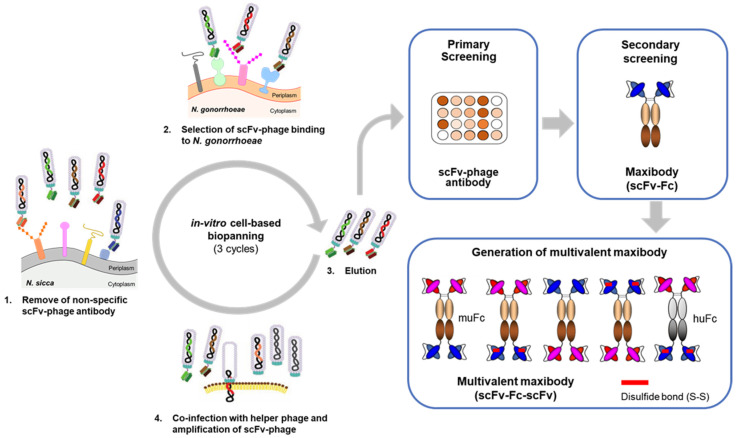
Overview of bio-panning and isolation of *Neisseria gonorrhoeae* specific antibodies. (**Left**) Selection of *N. gonorrhoeae*-specific antibody via in-vitro cell-based bio-panning using phage display technique: Step 1, depletion of the scFv-phage Ab library with *N. sicca* to remove gonorrhea-unrelated scFv-phage antibodies; Step 2, incubation of the depleted scFv-phage Ab library with *N. gonorrhoeae*; Step 3, elution of *N. gonorrhoeae*-bound scFv-phage after removing the unbound scFv-phage antibodies; Step 4, recovery and amplification of *N. gonorrhoeae* scFv-phage antibodies using helper phage in *E. coli* TG1. (**Right**) After three rounds of bio-panning, individual scFv-phage antibodies were preliminarily screened by phage ELISA. The selected scFv-phage antibodies were converted to maxibody and multivalent maxibody and analyzed to identify the optimal *N. gonorrhoeae*-specific maxibody pair for diagnosis (see main text for details).

The scFv-phage antibodies (Abs) binding to *N. gonorrhoeae* were selected via in-vitro bio-panning on *N. gonorrhoeae* isolates using human naïve scFv-phage antibody libraries, following the method we previously described, with minor modifications [41]. The overall bio-panning on *N. gonorrhoeae* cells is shown schematically in Figure 1. Briefly, scFv-phage antibody libraries (10^11^ CFU) depleted with *N. sicca* were incubated with *N. gonorrhoeae* clinical isolates to obtain *N. gonorrhoeae*-specific scFv-phage antibodies. According to standard methods, the eluted scFv-phage was propagated in *E. coli* TG1 cultures under the log-phase and rescued using VCSM13 helper phage [42,43]. A total of three rounds of selection were carried out using the amplified scFv-phage from the previous rounds as the input phage, with a gradual increase in the number of washes using PBS containing 0.1% Tween 20 (PBST).

### 2.4. Screening of scFv-Phage by Phage ELISA

To analyze scFv-phage antibody binding to *N. gonorrhoeae*, phage ELISA was performed using the standard protocol described in the literature [42,44]. Briefly, individual TG1 clones harboring scFv-phage Abs from the last round of selection were grown to the log-phase. The cells infected with VCSM13 were grown overnight at 25 °C to produce scFv-phage Abs. Phage ELISA was performed by coating Maxisorp plates (Nunc, Waltham, USA) with 1 µg/mL of *Neisseria* cell lysates in 50 mM NaHCO_3_ at pH 8. After blocking with PBST containing 3% skim milk (MPBS), the scFv-phage was added to the plates and incubated at room temperature (RT) for 1 h. Bound scFv-phage was detected with mouse horseradish peroxidase (HRP)-conjugated anti-M13 antibody (GE Healthcare, Chicago, USA) and HRP substrate at 450 or 405 nm using Synergy H1 Microplate Reader (BioTek, Winooski, VT, USA).

### 2.5. Conversion of scFv-Phage Antibodies into Maxibodies and Multivalent Maxibodies

The scFv fragment derived from the scFv-phage Abs was sub-cloned into the pCEP-4 mammalian expression vector (Invitrogen, Carlsbad, USA) using AgeI and HindIII restriction sites to generate scFv fused with the Fc fragment of mouse or human IgG1 to produce scFv-Fc (maxibody) as shown in Figure 1. The scFv gene inserts were amplified by polymerase chain reaction (PCR) with primers:

5′-GCCCAGACCGGTGAGGTGCAGCTG-3′ and

5′-GTGATGGTGCTGGCCAAGCTTGCCTAGGAC-3′.

Multivalent clone-1/4 maxibody and a disulfide-linked multivalent clone-1/4 maxibody (clone-1/4(ds)) were derived from the antibody Clone 1 maxibody to which the (Gly4Ser)2 linker and the scFv domain of antibody Clone 4 were fused at the C-terminus of the maxibody using AflII and NheI restriction sites (Figure S2). The scFv2 fragments were obtained for sub-cloning by PCR using primers:

5′-AATGGCCCAGGCGGCCCTTAAGGAGGTGCAGCTG-3′ and

5′-TAATTAGGCCCGGCCTGGCTAGCCCCTAGGACCGT-3′.

The disulfide bond designated “ds”, as in multivalent clone-1/4 (ds) maxibody, was generated via cysteine residues incorporated into V_H_44 and V_L_100 by point mutations to replace the glycine residues [45].

Maxibodies and multivalent maxibodies were transiently expressed in HEK293F and purified using MabSelect SuRe affinity chromatography as previously described, with minor modifications [41]. The concentrations of the purified maxibodies were determined by measuring the absorbance at 280 nm, using the calculated extinction coefficient based on the amino acid sequence of the maxibody [46]. The purify of the maxibodies was analyzed by sodium dodecyl sulfate-polyacrylamide gel electrophoresis (SDS-PAGE).

### 2.6. Binding Analysis by ELISA

Analysis of maxibody binding to *Neisseria* was performed by ELISA as described for phage ELISA above, with minor modifications. Commercially available mouse A30-Ab1 and A30-Ab2 anti-*N. gonorrhoeae* Abs (hereafter referred to as control Ab1 and Ab2) (Artron BioResearch, Burnaby, Canada) were used to compare with the in-house generated Abs. Goat HRP-conjugated anti-mouse Fc-specific antibody was used for the detection of maxibody and multivalent maxibody bound to *Neisseria*. An irrelevant mouse isotype control was used as the negative control antibody.

Sandwich ELISA was also performed to analyze multivalent maxibody binding to *N. gonorrhoeae* using 10 nM maxibody and multivalent maxibody. To capture the antigen, maxibody Clone 4, Clone-1/4, or Clone-1/4(ds) fused to mouse Fc (muFc) were immobilized on a Maxisorp plate. *Neisseria* lysates were added and incubated to allow binding to the maxibody or multivalent maxibody. *Neisseria* bound to the capturing maxibodies was detected by adding multivalent maxibody or clone-1/4(ds) fused to human Fc (huFc) at a final concentration of 10 nM, followed by incubation for 1 h. The bound Abs were detected with goat HRP-conjugated anti-human Fc-specific antibody and tetramethylbenzidine substrate. The absorbance at 450 or 405 nm was measured using a microplate reader. The *t*-test was applied using Prism 8 (GraphPad software). The diagnostic sensitivity and 95% CI values for detecting *N. gonorrhoeae* were calculated using MedCalc statistical software [47]. Briefly, sensitivity, a measure of how well the assay correctly identified the proportion of true *N. gonorrhoeae* positives tests out of all patient-derived *N. gonorrhoeae* was calculated as follows:

% Sensitivity = [number of positive isolate detected/number of total positive isolates] × 100%

### 2.7. Determination of Maxibody Affinity by ELISA

The affinity of the maxibodies was determined by ELISA as described above, except that a fixed concentration (1 μg/mL) of *N. gonorrhoeae* WHO L was immobilized on the plates and incubated with 2-fold serial dilutions of maxibody starting from 10 µg/mL. The bound maxibodies were detected with goat HRP-conjugated anti-mouse Fc-specific antibody and HRP substrate. The absorbance at 450 nm was measured using a microplate reader, and the percent binding for each maxibody was calculated using the formula: 100%  ×  [(A_450_ at each concentration of maxibody)/(A_450_ at 10 µg/mL maxibody)]. The half-maximal effective concentration (EC_50_) values were determined using GraphPad Prism 8.

## 3. Results and Discussion

### 3.1. Molecular Characterization and Antimicrobial Susceptibility of N. gonorrhoeae Clinical Isolates

The molecular epidemiological typing of *N. gonorrhoeae* is crucial for monitoring the spread of *N. gonorrhoeae* strains [38]. Eleven clinical isolates of *N. gonorrhoeae* were subjected to NG-MAST and porB sequence typing using PubMLST (Table 1). Ten different sequence types (STs) were identified from the 11 *N. gonorrhoeae* isolates. In particular, clinical isolate #1481 was identified as a novel ST, whereas the clinical isolates #832 and #1167 contained the same STs. Analysis of the antimicrobial susceptibility of the *N. gonorrhoeae* isolates revealed a lack of resistance to third-generation antibiotics in the strains tested (Table S1). However, all strains showed resistance to ciprofloxacin antibiotics. In contrast, only the #1471 strain was sensitive to penicillin, whereas only the #1446 strain was resistant to the second-cephalosporin antibiotic cefuroxime. Interestingly, only two penicillin-resistant isolates (#832 and #1446) tested positive for β-lactamase production, which provides antibiotic resistance. Overall, a considerable genetic diversity was observed among the *N. gonorrhoeae* clinical isolates. Thus, we used these different *gonococci* for the selection of antibodies against *N. gonorrhoeae*.

### 3.2. Bio-Panning and Selection of scFv-Phage Antibodies against N. gonorrhoeae Strains

*N. gonorrhoeae* is a particularly clonal species with all isolates clustering tightly together [48]. Thus, to select antibodies against *N. gonorrhoeae*, in-vitro cell-based bio-panning was performed against the eight patient-derived isolates as depicted in Figure 1. After depleting the phage antibodies with *N. sicca* cells to remove non-specific binders, three rounds of bio-panning were carried out to isolate *N. gonorrhoeae*-specific antibodies. An approximately 15,400-fold increase in scFv-phage output was detected by the third round of bio-panning, indicating the successful enrichment of scFv-phage antibodies binding to *N. gonorrhoeae* cells (Table 2).

**Figure 2:**
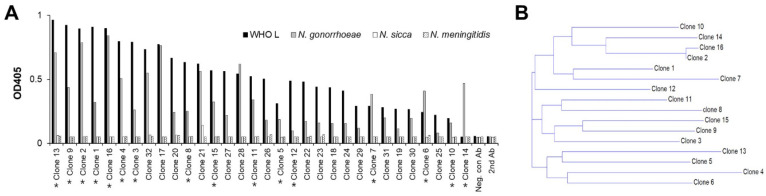
Identification of *N. gonorrhoeae*-specific scFv-phage clones. (**A**) The binding properties of randomly selected scFv-phage monoclonal antibodies to *N. gonorrhoeae* and WHO L (*N. gonorrhoeae* reference strain) were measured via throughput phage ELISA. Any scFv-phage antibodies binding to *N. sicca* and *N. meningitidis* were considered non-specific *N. gonorrhoeae* antibodies. Myo22 was used as the negative control for the scFv-phage. The unique anti-*N. gonorrhoeae* scFv-phage clones and secondary antibody (anti-M13-HRP) are indicated by a star (*) and 2nd Ab, respectively. (**B**) Phylogram tree for the comparison of scFv amino acid sequences in the 16 unique anti-*N. gonorrhoeae* scFv-phage clones. Phylogram trees were produced using the AlignX program, and the clone numbers are listed along the plot’s right side.

The specificity of the scFv-phage antibodies selected from the bio-panning was analyzed by phage ELISA (Figure 2A). Five hundred seventy-six individual clones randomly chosen from the final round of bio-panning against *N. gonorrhoeae* were examined for binding to eight *N. gonorrhoeae* isolates and cross-reactivity to *N. sicca* and *N. meningitidis*. We included *N. meningitidis* because it is major pathogenic *Neisseria* and more closely related to *N. gonorrhoeae* than other species within the genus [3,49].

As shown in Figure 2A, 30 scFv-phage clones binding to *N. gonorrhoeae* and WHO L (WHO *N. gonorrhoeae* reference strain L resistant to third-generation antibiotics) were selected with a binding ratio > 4 for WHO L to *N. sicca*. In addition, none of the 30 clones cross-reacted with *N. sicca* and *N. meningitidis*. The irrelevant anti-Myo22 scFv-phage antibody, the negative control phage antibody, did not react with any *Neisseria* strains tested to determine the sensitivity of the antibodies obtained via bio-panning. Diversity analysis of these 30 scFv-phage clones by nucleotide sequencing revealed 16 unique sequences (Figure 2B). These results demonstrate the successful selection of diversified *N. gonorrhoeae*-specific scFv-phage antibodies by direct bio-panning on cells.

### 3.3. Generation and Antigen-Binding Characterization of Anti-N. gonorrhoeae Maxibody

**Figure 3:**
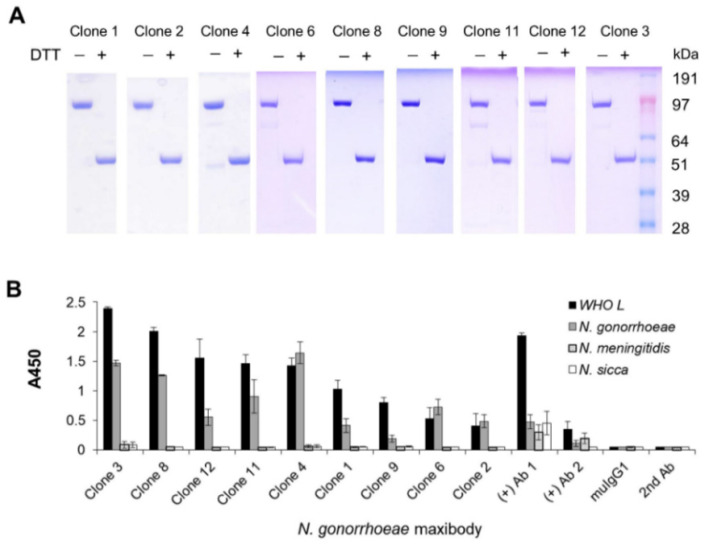
Generation and binding of anti-*N. gonorrhoeae* maxibody. (**A**) Purified maxibodies were resolved on 4–12% (*w*/*v*) bis-Tris SDS-PAGE under non-reducing (−DTT) and reducing (+DTT) (**B**) Analysis of monoclonal maxibody specificity of *N. gonorrhoeae* and WHO L reference strain. Commercially available anti-*N. gonorrhoeae* antibodies (Ab1 and Ab2) were used for comparison. Irrelevant mouse isotype control was included as the negative control antibody. Maxibodies Ab1 and Ab2 were detected with an HRP-anti-mouse antibody. The absorbance values are presented as the mean ± standard deviation (SD) of several independent experiments. DTT, dithiothreitol.

Based on the binding reactivity and scFv sequence diversity shown in Figure 2, nine of the unique scFvs with the highest *N. gonorrhoeae* and WHO L binding ratio compared to the gonorrhea-unrelated *N. sicca* and *N. meningitidis* strains were selected for further analysis. The scFv gene of the scFv-phage was sub-cloned to generate scFv fused with the mouse IgG_1_Fc domain (Maxibody) (Figure 1). Maxibodies were expressed transiently in HEK293F cells and purified using MabSelect SuRe™ affinity chromatography. Each of the purified *N. gonorrhoeae* maxibodies showed a molecular mass consistent with the predicted mass based on the primary structure under non-reducing (~104 kDa) and reducing conditions (~52 kDa) (Figure 3A). The binding specificity of the maxibodies for *Neisseria* strains was analyzed by ELISA and compared to the commercially available *N. gonorrhoeae* antibodies Ab1 and Ab2. As expected, all nine maxibodies showed specific binding to eight *N. gonorrhoeae* and the WHO L strain, but lacked cross-reactivity with *N. sicca* or *N. meningitides* (Figure S1 and Figure 3B). This result indicated that the conversion of the selected scFv-phage to its corresponding maxibody retained its binding specificity. In contrast, the positive controls Ab1 and Ab2 showed binding to *N. gonorrhoeae* strains and cross-reactivity with *N. sicca* and *N. meningitidis*, although Ab2 exhibited better specificity than Ab1. Moreover, both Ab1 and Ab2 showed lower binding intensity to *N. gonorrhoeae* than most of the maxibody clones based on absorbance at 450 nm. Moreover, maxibody Clones 1 and 4 detected most of the isolates (Figure S1). The higher sensitivity and specificity of the selected maxibody for the target antigen than the commercial Ab1 and Ab2 suggest that the maxibody can be used to diagnose gonorrheal infections.

### 3.4. Two Maxibodies Exhibited Binding against a Broad Spectrum of N. gonorrhoeae Strains

To determine whether the maxibody Clones 1 and 4 derived from the phage-display could detect *N. gonorrhoeae* in clinical settings, we further analyzed the sensitivity of maxibodies with eleven patient-derived *N. gonorrhoeae isolates* (*additional* # 834, 840 and 1167 isolates). By screening the nine maxibody clones against the clinical isolates listed in Table 1, the maxibodies were classified into five groups based on the proportion of the clinically positive isolates and the molecular characteristics of *N. gonorrhoeae* (Figure S1 and Table 3).

**Figure 4:**
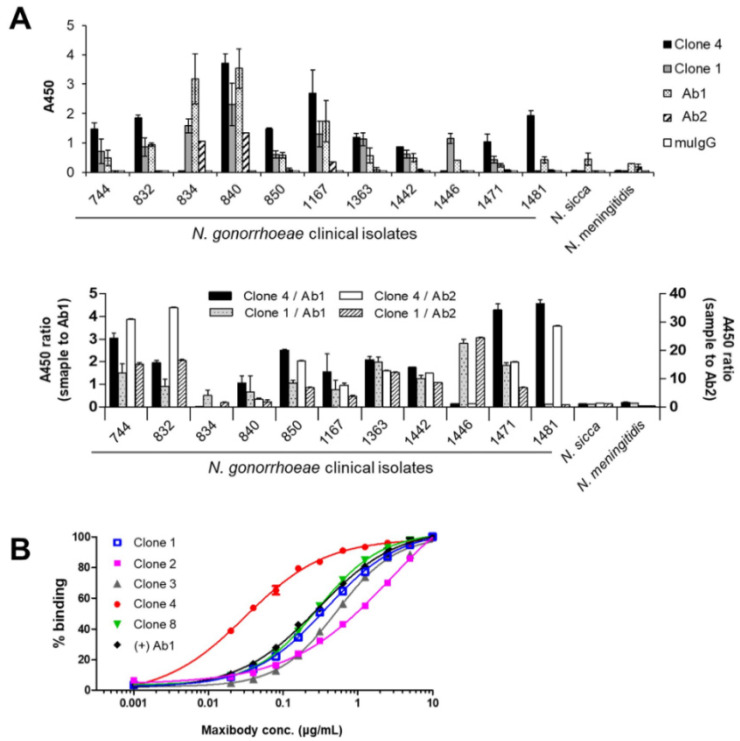
Binding characteristics of the maxibodies against a broad spectrum of *N. gonorrhoeae***.** (**A**) The binding of maxibody Clones 1 and 4 are representative of the maximum cross-reactivity with diverse *N. gonorrhoeae* strains (upper panel). The A450 ratio of maxibody to Ab1 (left *Y*-axis) and maxibody to Ab2 (right *Y*-axis) are shown in the lower panel. (**B**) The affinity of *N. gonorrhoeae* maxibodies was determined by ELISA using WHO L reference strains. The *N. gonorrhoeae* reference strains were immobilized and incubated with serially diluted maxibodies followed by reaction with an HRP-conjugated secondary antibody. The EC_50_ was calculated using GraphPad Prism. Clone 1 (Group 1); Clone 2 and 8 (Group 4); Clone 3 (Group 3); and Clone 4 (Group2).

Maxibody Clones 1 and 4 detected most clinical isolates and were assigned as groups 1 and 2, respectively (Table 3, Figure 4A). Maxibody Clone 1 recognized all, except *N. gonorrhoeae* isolate No. 1481, whereas maxibody Clone 4 showed binding to all, except *N. gonorrhoeae* isolates No. 834 and 1446, which suggests that maxibody Clones 1 and 4 had different binding sites (i.e., epitopes) on *N. gonorrhoeae.* The maxibodies in Groups 3 and 4 showed sensitivity up to 75% and 50%, respectively, toward the isolates, whereas maxibody Clone 9 and commercial Ab2 showed ~25%, the lowest sensitivity to the isolates. In contrast, commercial Ab1 showed binding against all 11 clinical isolates (Figure 4A). However, its significant cross-reactivity with gonorrhea-unrelated *N. sicca* and *N. meningitidis* strains is not a characteristic of preferred diagnostic antibodies.

The affinity of the maxibodies derived from the four groups was estimated by ELISA using a fixed concentration of WHO L immobilized on the plates and probed with 2-fold serial dilutions of the maxibodies (Figure 4B). The Clone 4 (Group 2 in Table 2) maxibody showed the highest affinity for the antigen with an EC_50_ value of 3 nM, which is a 6.3-fold increase in affinity compared to the control Ab1 (19 nM). In comparison, maxibody Clones 1 and 8 showed a similar affinity to the control Ab1. Thus, based on the sensitivity and affinity for *N. gonorrhoeae*, maxibody Clones 1 and 4 were selected to further evaluate their diagnostic value.

### 3.5. Generation of Multivalent N. gonorrhoeae Maxibody

**Figure 5:**
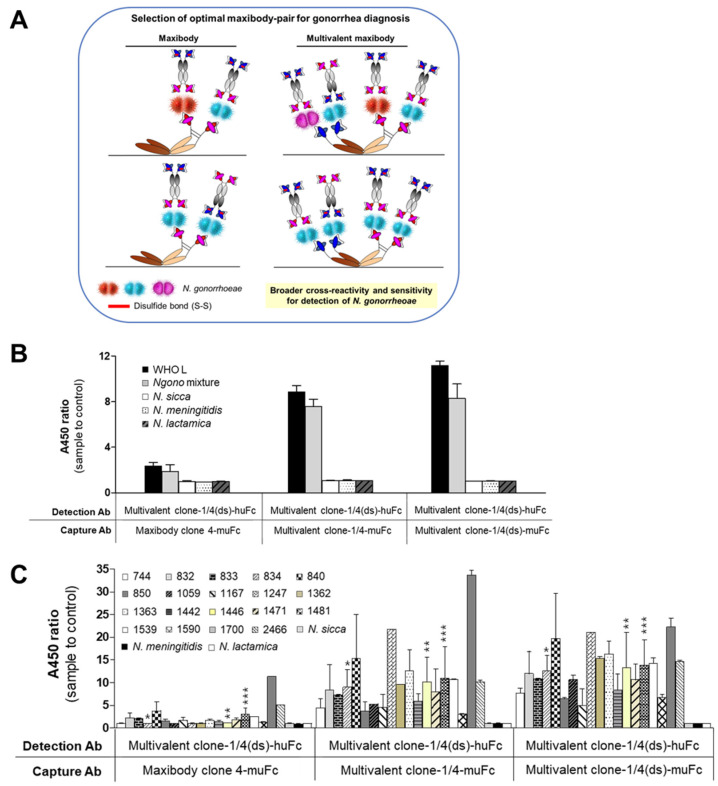
Analysis of the optimal maxibody pair for diagnosis. (**A**) A schematic presentation of sandwich ELISA for determining the optimal combination of capturing and detecting maxibodies for gonorrhea. Clone 4 maxibody containing the muFc domain, and multivalent maxibodies (Clone-1/4-muFc and Clone1/4(ds)-muFc) were used to capture *N. gonorrhoeae*. Multivalent Clone1/4(ds)-huFc maxibody detected the antigen bound to the capturing maxibody. (**B**) Identification of the optimal capturing maxibody using the WHO L reference strain and the mixture of *N. gonorrhoeae* isolates. *N. meningitidis*, *N. lactamica*, and *N. sicca* were used as controls for irrelevant *Neisseria* strains. (**C**) A broad-spectrum of patient-derived *N. gonorrhoeae* isolates was used as antigens for the antibodies. Multivalent clone-1/4(ds)-huFc was used as the detecting maxibody in conjunction with HRP-conjugated anti-huFc antibody. The A450 value obtained in the absence of antigen was used to determine the A450 ratio values expressed as the mean ± standard deviation (SD) of three independent experiments. The clinical isolates that were undetected by maxibody Clones 1 or 4 are indicated: Nos. 1481 (***), 834 (*), and 1442 (**).

Antibodies for immunodiagnostics should exhibit a broad-spectrum binding sensitivity with a high affinity to facilitate rapid diagnosis. The antigen-binding analysis of the *N. gonorrhoeae* maxibody clones with patient-derived isolates indicated that Clones 1 and 4 exhibited the maximum broad-spectrum for *N. gonorrhoeae* strains but with different epitopes on *N. gonorrhoeae*. The bifunctional and multivalent maxibody specific for *N. gonorrhoeae* strains should offer higher avidity effect and a wide range of clinical applications with improved immunoassays. Thus, to further evaluate whether these antibodies could be applied to diagnose with high sensitivity and specificity, we generated a multivalent maxibody with bifunctional epitopes using Clones 1 and 4 to maximize antigen sensitivity and enhance the signal-to-noise ratio for a lower antigen detection limit. Specifically, clone-1/4 and clone-1/4(ds), multivalent maxibodies in a scFv1-Fc-scFv4 format, were constructed for tetravalent binding to two different epitopes on *N. gonorrhoeae* (Figure S2 and Figure 5A). The scFv domains were fused to the immunoglobulin huFc or muFc domain with or without a disulfide bond designated “ds”, as in clone-1/4(ds) to stabilize the scFv domain. The molecular weight of the purified multivalent maxibodies was consistent with the expected mass for reduced (79 kDa) and non-reduced (158 kDa) antibodies based on the amino acid composition (Figure S3).

### 3.6. Selection of the Optimal Maxibody Pair for Immunodiagnostic Applications

Immunodiagnostic methods use an antibody pair for detecting the antigen of interest with one antibody, while the other antibody quantifies the antigen captured on the pairing antibody [50]. For example, immunoassay-based POCTs use two antibodies referred to as capture and detection antibodies, which bind to different sites on the antigen. Therefore, it is imperative to select the optimal capture-detection maxibody pair for immunodiagnostic applications [51]. We determined the optimal pair of *N. gonorrhoeae* maxibodies for sandwich ELISA by evaluating the possible combinations of capturing and detecting antibodies. Multivalent maxibodies clone-1/4-muFc and clone-1/4(ds)-muFc, and maxibody Clone 4-muFc were used as the antigen-capturing maxibodies, whereas huFc-tagged multivalent clone-1/4(ds) was used as the detection maxibody (Figure 5A). Clone 1 was not considered as the antigen-capture antibody for rapid diagnostics due to its lower affinity for *N. gonorrhoeae* than Clone 4. The efficiency of maxibodies as capture antibody was evaluated using the WHO L reference strain and the *N. gonorrhoeae* clinical isolate mixture. As shown in Figure 5B, the multivalent maxibodies specifically detected both the WHO L reference strain and the *N. gonorrhoeae* mixture. The multivalent maxibodies did not react with the irrelevant *N. meningitides*, *N. sicca*, and *N. lactamica* strains. Further, multivalent clone-1/4 and clone-1/4(ds), when used as the antigen capture maxibodies, showed at least a four-fold increase in binding intensity for detecting *N. gonorrhoeae* compared to antigen capture using the Clone 4 maxibody. However, clone-1/4-muFc and clone-1/4(ds)-muFc showed almost similar efficiency of *N. gonorrhoeae* antigen-capture indicating that the target specificity of each scFv domain of the clones was retained in the multivalent maxibody, resulting in enhanced avidity for and intrinsic binding to *N. gonorrhoeae*.

The sensitivity of *N. gonorrhoeae* multivalent maxibodies for antigen detection in the 19 patient-derived isolates was also evaluated by sandwich ELISA. The multivalent maxibody clone-1/4 and clone-1/4(ds) showed increased binding intensity for all gonorrhea-positive isolates in a range of 2- to 20-fold higher than *N. gonorrhoeae* antigen-capture with maxibody Clone 4 (Figure 5C). More importantly, the multivalent maxibodies facilitated the detection of all the clinical isolates, including the previously undetected isolates with maxibody Clones 1 and 4. Furthermore, the confidence interval of multivalent maxibodies for the detection of *N. gonorrhoeae* increased to 82–100% compared to its corresponding maxibody Clone 1 or Clone 4 alone (Table 3). This finding was consistent with our hypothesis that multivalent maxibodies promote stronger antigen binding and additional antigen recruitment via increased avidity and broader spectrum of *N. gonorrhoeae* strain specificity. Thus, the increased sensitivity and specificity of multivalent maxibodies facilitated the detection of diverse strains of *N. gonorrhoeae* and thus, potential application of multivalent maxibodies for diagnosis of gonorrheal infections.

## 4. Conclusions

The increased specificity and affinity of the antibody for its antigen is a desirable feature of reagents in immunological method-based POCTs. Recently, the use of an antibody tagged with alkaline phosphatase in conjunction with thionicotinamide-adenine dinucleotide (thio-NAD) for developing a sensitive immunoassay has been reported for the diagnosis of mycobacterium tuberculosis [52]. The multivalent maxibody platform, scFv-Fc-scFv, represents an excellent option for diagnosing gonorrheal infections.

In this study, we selected two maxibody Clones 1 and 4, with varying epitopes and recognition of broad strains of *N. gonorrhoeae* with high specificity and affinity. Although we did not extensively test for other *Neisseria* species, a severely pathogenic *N. meningitidis* and two non-pathogenic strains *N. lactamica* and *N. sicca* were included to evaluate the cross-reactivity of the *N. gonorrhoeae* maxibodies. Both Clones 1 and 4 maxibodies did not cross-react with negative control *Neisseria* species that are most closely related to *N. gonorrhoeae*. We demonstrated the superiority of multivalent scFv-1/4 maxibody in improving the sensitivity, CI, and detection limit compared to commercial Abs (Ab1 and Ab2) and the Abs currently in development for POCTs. The bispecific characteristic of the multivalent maxibodies provides additional specificity for *N. gonorrhoeae*. Therefore, we do not expect our *N. gonorrhoeae* maxibodies to cross-react with other *Neisseria* species but do cross-react with other *N. gonorrhoeae* isolates (i.e., broad-binding to *N. gonorrhoeae* strains). Our study suggests for the first time the potential application of the multivalent scFv-Fc-scFv maxibody platform for the sensitive detection of other infectious diseases and cancer.

Moreover, *N. gonorrhoeae* multivalent maxibodies were developed as a possible resolution to the unmet diagnostic sensitivity of immunodiagnostics for gonorrheal infections. The limitation of this study was the use of relatively small numbers of *N. gonorrhoeae* isolates because this study focused on the development of a sensitive and specific multivalent maxibody for *N. gonorrhoeae* bacteria. For the clinical usefulness of a multivalent maxibody as an improvement for immunological diagnostic methods, the evaluation of a larger number of clinical specimens and additional contrast strains of *Neisseria* species remains an important aim of our follow-up study. Thus, further evaluation in the clinical setting and optimization of *N. gonorrhoeae* multivalent maxibody pairs are warranted to determine the clinical application for the disease-specific and sensitive detection of gonorrheal infections.

## Figures and Tables

**Table 1:** Molecular characteristics of *N. gonorrhoeae* clinical isolates.

NG Clinical Isolate #	NG-MAST ^1^			PubMLST ^2^
	Allele		Sequence Type (ST)	Allele
	*porB*	*tbpB*		*porB*
744	543	899	4278	12
832	4016	33	6734	18
834	1785	2422	15,525	8
840	4623	455	7693	632
850	8061	33	13,973	18
1167	4016	33	6734	18
1363	2514	455	11,361	8
1442	8521	60	14,668	11
1446	3764	110	12,402	8
1471	785	60	3611	12
1481	1659	1058	New ^3^	8

^1^ NG-MAST, *Neisseria gonorrhoeae* multiantigen sequence typing; ^2^ PubMLST, public multilocus sequence typing; ^3^ the sequence was not found in the database.

**Table 2:** Selective enrichment of scFv-phages by bio-panning.

Round of Screening	Input (CFU) ^a^	Output (CFU) ^b^	Enrichment Fold	Total Enrichment Fold
1	1 × 10^11^	6.6 × 10^4^	1	N/A
2	1 × 10^11^	3.25 × 10^5^	4.9	4.9
3	1 × 10^11^	5 × 10^9^	15,384	75,382

^a^ Number of CFU (colony forming units) of scFv-phages incubated with *N. gonorrhoeae*. ^b^ Total number of CFUs of scFv-phages in eluates at the end of bio-panning round.

**Table 3:** Characterization of maxibodies according to Ab specificity for clinical isolates.

Ab			No. of Positive Isolates Detected (% Sensitivity) ^1^	95% CI	Cross Reactivity with Control *Neisseria* spp.	Clinical Isolate No.
Maxibody	Group 1	Clone 1	10/11 (90.9%)	59 to 100	No ^2^	744, 832, 834, 840, 850, 1167, 1363, 1442, 1446, 1471
	Group 2	Clone 4	9/11 (81.8%)	48 to 98	No	744, 832, 840, 850, 1167, 1363, 1442, 1471, 1481
	Group 3	Clone 3	6/8 (75%)	35 to 97	No	832, 850, 1363, 1442, 1446, 1471
	Group 4	Clones 2, 6, 8, 11, 12	4/8 (50%)	16 to 84	No	832, 850, 1442, 1471
	Group 5	Clone 9	2/8 (25%)	3 to 65	No	850, 1363
Multivalent maxibody	Clone-1/4		19/19 (100%)	82 to 100	No	744, 832, 833, 834, 840, 850, 1059, 1167,1247, 1362, 1363, 1442, 1446, 1471, 1481, 1539, 1590, 1700, 2466
	Clone-1/4(ds)		19/19 (100%)	82 to 100	No	744, 832, 833, 834, 840, 850, 1059, 1167, 1247, 1362, 1363, 1442, 1446, 1471, 1481, 1539, 1590, 1700, 2466
Commercial Ab (Artron BioResearch)	A30-Ab1		11/11 (100%)	72 to 100	*N. sicca* and *N. meningitidis*	744, 832, 834, 840, 850, 1167, 1363, 1442, 1446, 1471, 1481
	A30-Ab2		3/11 (27.3%)	6 to 10	*N. sicca* and *N. meningitidis*	834, 840, 1167

^1^ The sensitivity and 95% CI values for detecting *N. gonorrhoeae* were calculated by MedCalc statistical software [47]. ^2^ No, no cross-reactivity was observed with *N. sicca*, *N. meningitidis*, and *N. lactamica*.

## Data Availability

Data sharing is not applicable to this article.

## Supplementary Materials

The following are available online at https://www.mdpi.com/2218-273X/11/3/484/s1, Table S1: Antimicrobial susceptibility of *Neisseria gonorrhoeae* clinical isolates, Figure S1: binding characteristics of maxibodies against a broad spectrum of *N. gonorrhoeae*, Figure S2: construction of *N. gonorrhoeae* multivalent maxibody, Figure S3: purification of *N. gonorrhoeae* multivalent antibody.
