# Supplementary material for: Neisseria gonorrhoeae Multivalent Maxibody with a Broad Spectrum of Strain Specificity and Sensitivity for Gonorrhea Diagnosis
Source: Biomolecules. 2021 Mar 23;11(3):484. doi: 10.3390/biom11030484 (PMC8004885; doi:10.3390/biom11030484)
Supplement: Supplementary file 1 [file biomolecules-11-00484-s001.pdf]

biomolecules

MDPI

Supplementary material

Neisseria gonorrhoeae Multivalent Maxibody with a Broad Spectrum of Strain Specificity and Sensitivity for Gonorrhea Diagnosis

Jieun Jeong [1,†], Jae-Seok Kim[2,3,†], Junghyeon Lee[4], Yu Ri Seo[5], Eugene C. Yi[5,*] and Kristine M. Kim[1,4*]

1 Division of Biomedical Convergence, College of Biomedical Science, Kangwon National University, Chuncheon, Gangwon, 24341, Republic of Korea; jje7442@kangwon.ac.kr (J.J.)
2 Department of Laboratory Medicine, Kangdong Sacred Heart Hospital, Gangdong-gu, Seoul, 05355, Republic of Korea; jaeseok@hallym.ac.kr (J.S.K.)
3 Department of Laboratory Medicine, Hallym University, College of Medicine, Chuncheon, Gangwon, 24341, Republic of Korea
4 Department of Bio-Health Convergence, Kangwon National University, Chuncheon, Gangwon, 24341, Republic of Korea; sunshine2960@kangwon.ac.kr (J.L.)
5 Department of Molecular Medicine and Biopharmaceutical Sciences, Graduate School of Convergence Science and Technology and College of Medicine or College of Pharmacy, Seoul National University, Seoul, 03080, Republic of Korea; yuri.seo@snu.ac.kr (Y.S.)

† These authors contributed equally to this work.
* Correspondence: euyi@snu.ac.kr; Tel.: +82-2-740-8926; Fax: +82-2-3673-2167 (E.C.Y.); kmkim@kangwon.ac.kr; Tel.: +82-33-250-8382; Fax: +82-33-250-8382 (K.M.K.)

Table S1. Antimicrobial susceptibility of *Neisseria gonorrhoeae* clinical isolates

**Antibiotics**		***N. gonorrhoeae* clinical isolates**							
**Chemical groups**	**Drugs**	**744**	**832**	**850**	**1363**	**1442**	**1446**	**1471**	**1481**
Penicillins	Penicillin	I	R	R	I	I	R	S	R
Aminoglycosides	Spectinomycin	n.d.	n.d.	n.d.	S	S	S	S	S
Tetracyclines	Tetracycline	R	R	R	I	R	R	R	R
Quinolones	Ciprofloxacin	R	R	R	R	R	R	R	R
2nd generation cephalosporins	Cefuroxime	S	S	S	S	S	R	S	S
3rd generation cephalosporins	Cefixime	n.d.	n.d.	S	S	S	S	S	S
	Cefotaxime	S	S	S	n.d.	n.d.	S	n.d.	S
	Ceftriaxone	n.d.	n.d.	n.d.	S	S	n.d.	S	n.d.

S, susceptible; I, intermediate susceptible; R, resistant; n.d., not determined
[a]β-lactamase is not antibiotics. It is a genetic determinants of resistance to beta-lactam antibiotics.

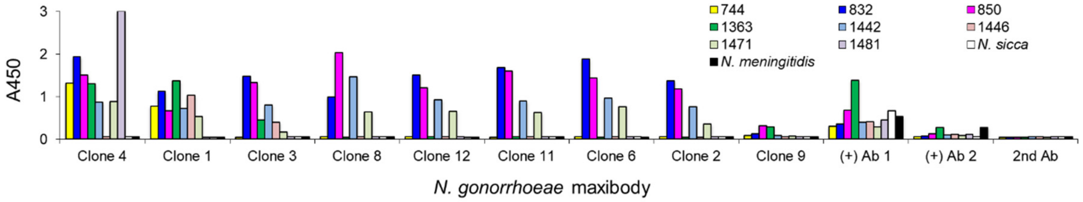

Figure S1. Binding characteristics of the maxibodies against a broad spectrum of *N. gonorrhoeae*. Nine maxibodies were analyzed for binding against various *N. gonorrhoeae* isolates. Commercially available anti-*N. gonorrhoeae* antibodies (positive control Ab1 and Ab2) were used for comparison. Irrelevant mouse isotype control was included as the negative control antibody. The antibodies were detected with an HRP-anti-mouse antibody.

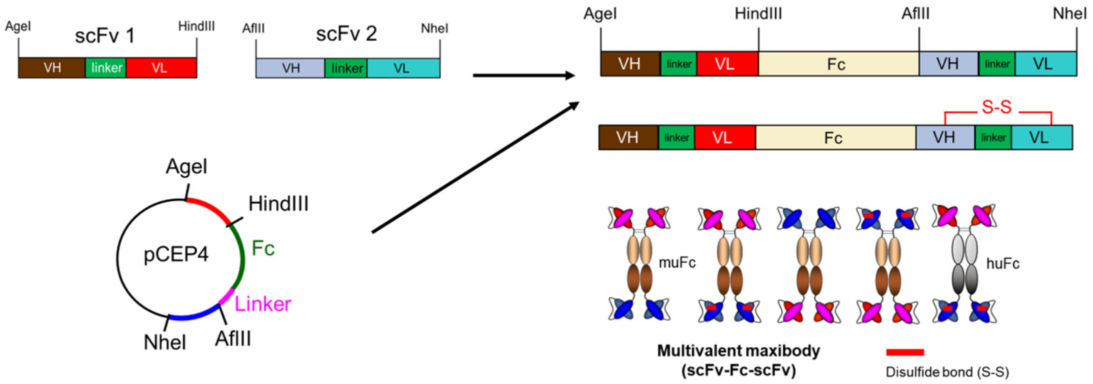

Figure S2. Construction of *N. gonorrhoeae* multivalent maxibody. The multivalent maxibody containing the disulfide bond formed between V_H and V_L is indicated by S-S and the red line in the schematic presentation. The scFv1 insert genes were amplified with primers by PCR for 30 cycles consisting of denaturation at 95 °C for 30 sec, annealing at 63 °C for 30 sec, and elongation at 72°C in a cycle. The scFv2 fragments for sub-cloning were obtained by PCR with 10 cycles of 95 °C for 1 min, 65 °C for 1 min, 72 °C for 1 min, followed by 20 cycles of 95 °C for 1 min, and 72 °C for 3 min with a final 10 min extension at 72 °C. See Materials and Methods in the main text for details.

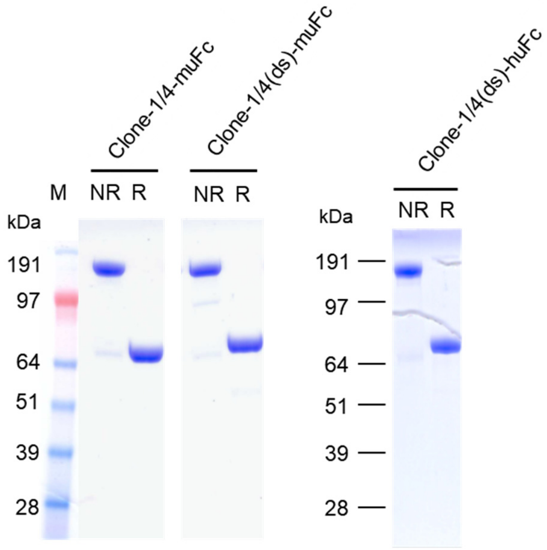

Figure S3. SDS-PAGE analysis of *N. gonorrhoeae* multivalent antibody. Purified antibodies were resolved on 4-12% (w/v) bis-Tris SDS-PAGE under non-reducing (NR) and reducing (R) conditions. Protein bands were visualized by staining with Coomassie Brilliant blue. The molecular weight of the purified multivalent maxibodies was consistent with the expected mass for reduced (79 kDa) and non-reduced (158 kDa) based on the amino acid composition.
